# GRK5 deficiency exaggerates inflammatory changes in TgAPPsw mice

**DOI:** 10.1186/1742-2094-5-24

**Published:** 2008-06-03

**Authors:** Longxuan Li, Jun Liu, William Z Suo

**Affiliations:** 1 Lab. for Alzheimer's Disease & Aging Res., VA Med. Center, Kansas City, MO 64128, USA; 2 Department of Neurology, Univ. of Kansas Med. Center, Kansas City, KS 66170, USA; 3 Department of Molecular & Integrative Physiology, Univ. of Kansas Med. Center, Kansas City, KS 66170, USA; 4 Department of Neurology, The 2nd Affiliated Hospital, Sun Yat-sen University, Guangzhou, 510120, PR China

## Abstract

**Background:**

Deficiency of membrane G-protein coupled receptor (GPCR) kinase-5 (GRK5) recently has been linked to early AD pathogenesis, and has been suggested to contribute to augmented microglial activation *in vitro *by sensitizing relevant GPCRs. However, GRK5 deficient mice did not show any signs of microgliosis, except for their moderate increase in axonal defects and synaptic degenerative changes during aging. We have speculated that one possible reason for the absence of microgliosis in these animals might be due to lack of an active inflammatory process involving activated GPCR signaling, since GRKs only act on activated GPCRs. The objective of this study was to determine whether the microgliosis is exaggerated in TgAPPsw (Tg2576) mice also deficient in GRK5, in which fibrillar β-amyloid (Aβ) and an active inflammatory process involving activated GPCR signaling are present.

**Methods:**

Both quantitative and qualitative immunochemistry methods were used to evaluate the microgliosis and astrogliosis in these animals.

**Results:**

We found that inactivation of one copy of the GRK5 gene in the TgAPPsw mice resulted in approximately doubled extent of microgliosis, along with significantly exaggerated astrogliosis, in both hippocampus and cortex of the aged animals. Consistent with previous observations, the activated microglia were located primarily near or surrounding the fibrillar Aβ deposits.

**Conclusion:**

The results demonstrate that GRK5 deficiency *in vivo *significantly exaggerates microgliosis and astrogliosis in the presence of an inflammatory initiator, such as the excess fibrillar Aβ and the subsequent active inflammatory reactions in the TgAPPsw mice.

## Background

Alzheimer's disease (AD) is a devastating neurodegenerative disorder affecting a growing number among the aging population. Although its etiology remains to be elucidated, pathological evidence indicates that, in addition to the characteristic senile plaques and neurofibrillary tangles, significant brain inflammatory changes, featured with exaggerated gliosis and increased proinflammatory cytokines, are an important component of the pathology [1,2].

Inflammation is a double-edged sword. For instance in AD, an appropriate degree of inflammatory reaction may be beneficial for clearance of aggregated proteins and/or degenerated cells. However, if the inflammation is overly reactive, the secondary inflammatory neuronal damage may become a driving force for the disease, as appears to be the case in AD [1]. Therefore, it is important to understand what factors may exaggerate the brain inflammation in AD so that corresponding therapeutic approaches can be developed to ameliorate such changes.

The extent of inflammatory reactions may be regulated by a variety of factors, such as proinflammatory cytokines, chemokines, complement systems, and others. In AD, fibrillar β-amyloid (Aβ) has been shown to directly activate microglial cells and result in increased levels of proinflammatory cytokines, such as interleukin-1 beta (IL-1β) and tumor necrosis factor-α [1-3]. In addition, it can activate the complement system to generate C3a and C5a [4]. Moreover, fibrillar Aβ accumulation is associated with increased levels of many chemokines and chemokine receptors, such as monocyte chemoattractant protein-1 (CCR2 ligand), macrophage inflammatory protein 1 (CCR3 ligand), interferon-inducible protein-10 (CXCR3 ligand), IL-8 (ligand for CXCXR1 and CXCR2) and CCR1/3/5. [5-7]. Amongst the inflammatory regulators associated with AD, there is one large category that exerts their cellular effects through heterotrimeric G-protein-coupled receptors (GPCRs). These include all the chemokines [6] and the anaphylatoxins C3a and C5a [8,9].

In a recent study, we showed that deficiency of membrane GPCR kinase-5 (GRK5) occurs during early AD, and the GRK5 deficiency contributes to augmented microglial activation *in vitro *via impaired sensitization of relevant GPCRs [10]. GRK5 is one of seven GRK family members, whose primary function is to desensitize activated GPCRs by phosphorylating the activated receptor to promote uncoupling of receptor-dependent G-protein activation and by initiating receptor internalization [11,12]. Therefore, the lack of membrane (functional) GRK5 resulted in prolonged signaling and enhanced microglial activation, which well explained the phenomena that we observed in the cultured microglial cells [10]. Moreover, we speculated that the same phenomena might also take place *in vivo*. However, our later study in GRK5 deficient mice (GRK5KO), an animal model resembling the functional GRK5 deficiency in AD, failed to show any sign of microgliosis, except for moderate increase in axonal defects and synaptic degenerative changes in these animals [13]. The negative *in vivo *data appeared to be inconsistent with our earlier *in vitro *data. Nonetheless, a hallmark of GRK action is to function only after GPCRs are activated [11,12]. In other words, whether or not an impact of GRK5 deficiency on a particular stimulus can be revealed depends on whether or not such a stimulus is present. We have reported that, although the GRK5 deficiency indeed caused a mild increase of Aβ level, no fibrillar Aβ deposits or any significant signs of microglial activation were observed in the GRK5KO mice [13]. Therefore, we speculated that one reason for the absence of microgliosis in these animals might be due to lack of inflammatory initiators, such as fibrillar Aβ, or any of the downstream chemokines or anaphylatoxins.

In this study, we crossbred the GRK5KO mice with TgAPPsw (Tg2576) mice. TgAPPsw mice overexpress human β-amyloid precursor protein (βAPP) carrying Swedish double mutations, and have been shown to display fibrillar Aβ deposition and moderate levels of inflammation [14-16]. Therefore, the resultant TgAPPsw mice deficient in GRK5 should provide an ideal model to determine if our previous speculations are correct, and GRK5 deficiency plays a significant role in enhancing brain inflammation in AD.

## Methods

### Animals

GRK5KO mice were generated by targeted deletion of exons 7 and 8 of the GRK5 gene, encoding critical sub-elements I through III of the protein kinase catalytic domain, as detailed previously [17]. Heterozygous GRK5KO mice (C57/BL6 background) were bred with TgAPPsw (Tg2576) mice (also with a C57/BL6 background) to produce wild type (WT, APPsw^-/-^/GRK5^+/+^, *n *= 6), GRK5KO (APPsw^-/-^/GRK5^+/-^, *n *= 6), TgAPPsw (APPsw^+/-^/GRK5^+/+^, *n *= 6), and the double (APPsw^+/-^/GRK5^+/-^, *n *= 6) mice for this study. Due to known gender difference [18-20] [Li, 2008, BRB-submitted], only female mice (18-month old) were used in this study. A tail DNA preparation was used for genotyping as described below. All procedures for using these animals were approved by the KCVAMC Institutional Animal Care and Use Committee.

### Genotyping

**Figure 1:**
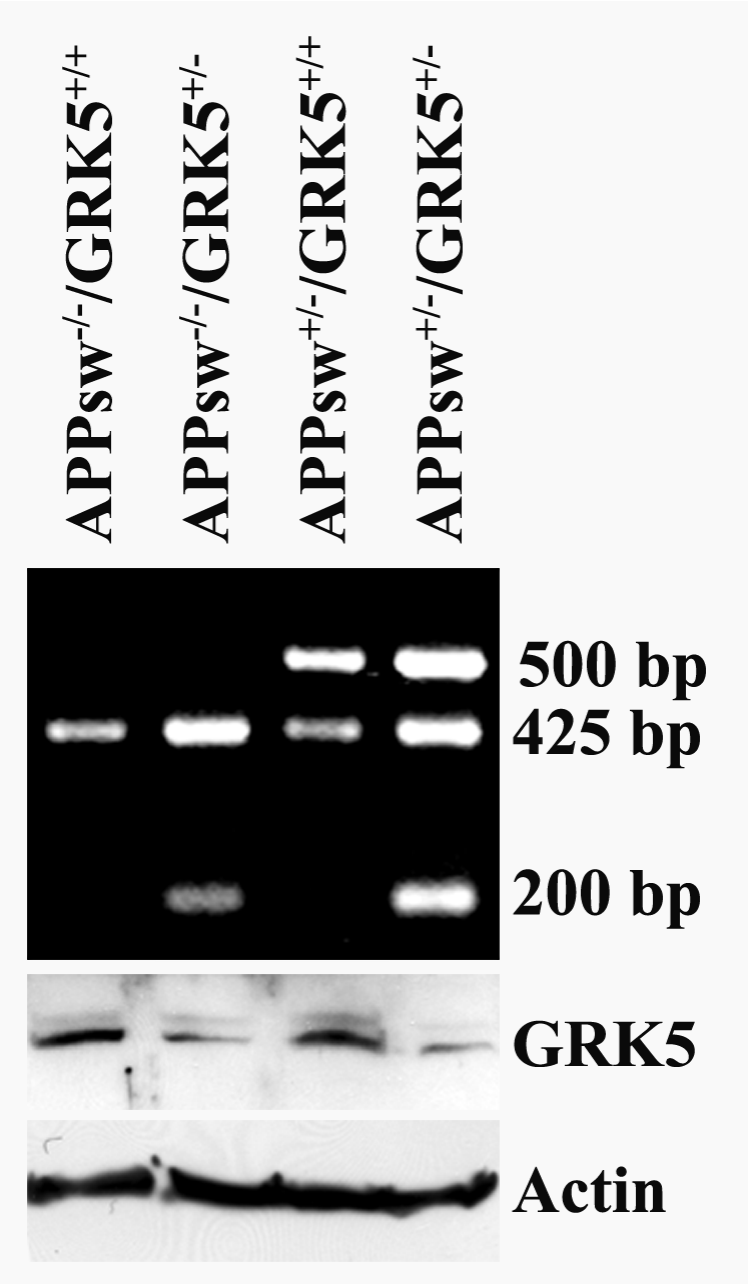
**Genotypes and GRK5 expression in TgAPPsw mice with reduced GRK5**. Top panel, an example of the genotyping results for the WT (APPsw^-/-^/GRK5^+/+^), heterozygote GRK5KO (APPsw^-/-^/GRK5^+/-^), TgAPPsw (APPsw^+/-^/GRK5^+/+^), and the double mice (APPsw^+/-^/GRK5^+/-^) using the ascribed five primers. 500 base pair (bp) band, human APPsw; 425 bp band, WT GRK5; 200 bp band, inactivated GRK5. Bottom panels are representative Western blots for GRK5 and β-actin (internal control for the amounts of sample loaded), respectively, as indicated.

Mouse-tail DNA was prepared as previously described [13]. Genotyping was performed by polymerase chain reaction (PCR) amplification of the tail genomic DNA with five different primers together: two human APP-specific primers, 5'CGGAGGAGGATGACTCGGAT3' and 5'CAGCTGCTGTCTCTCGTTGG3', which amplify a 500 bp DNA fragment, were used to identify the presence of the human APP transgene; three additional primers specific for WT murine GRK5 (425 bp) and the GRK5KO loci (200 bp) (5'CAAGTGTGAGGTAGGGTACAGAAT3', 5'CTATCCATTCACCTCCATGCTCCC3', and 5'AACTCTGGTACAGACAGGATCTCT3') were used to identify the presence of WT or the targeted murine GRK5 gene. The PCR used a 20 μl reaction volume containing 1.25 units of Flexi DNA polymerase (Promega, Madison, WI), 200 μM dNTPs, 2 mM MgCl_2_, and 250 nM of each of the APP preimers and 1 μM of each of the three GRK5 primers. The amplification protocol entailed 35 cycles of denaturation at 94°C for 15 s, annealing at 62°C for 15 s and extension at 72°C for 15 s, and then followed by a 10-min final extension at 72°C. The PCR products (10 μl) were then analyzed and visualized on 1.5% agarose gels. Fig. 1 shows an example of a typical genotyping result including the four different genotypes used in this study.

### Tissue preparations

All animals were anesthetized and perfused with cold phosphate buffered saline (PBS), their brains removed, and left hemispheres were post-fixed with 4% paraformaldehyde and processed for pathology. To minimize experimental variations, multi-brain embedding and sectioning techniques were adapted according to previously published procedures [13]. Briefly, twenty-four left hemispheres (including six from each genotype) were embedded into six blocks, with each block containing one hemisphere from each of the WT, GRK5KO, APP and the double mice, respectively. The multi-brain embedding allows the subsequent sectioning and staining procedures for all samples in a single block to be performed in a unified condition, which reduces variations that often occur when each brain is manipulated separately. Frozen sections were taken at a thickness of 20 μm in the coronal plane through the hemispheres. All sections were collected sequentially into eight groups with 160 μm intervals.

### Immunofluorescent (IF) staining

IF staining was performed on the multi-brain sections as previously described [13]. The only difference was the target antibody used: mouse monoclonal antibody (mAb) to human Aβ (Alpha Diagnostics International, San Antonio, TX; 1:600), rat mAb to CD45 (MCA1388, Clone: IBL-3/16, Serotec, Raleigh, NC; 1:25), rat mAb to mouse CD11b (Mac-1, Clone: M1/70.15, Serotec, Raleigh, NC; 1:25), mouse mAb to CD11b/c (Clone MRC OX-42, Serotec, Raleigh, NC; 1:25), rabbit polyclonal antibody (pAb) to human IL-1β (Santa Cruz Biotechnology, Santa Cruz, CA; 1:25), mAb to glial fibrillary acidic protein (GFAP) conjugated to Cy3 (Sigma, St. Louis, MO;1:200), or rabbit pAb to IL-6 (Pierce Biotechnology, Rockford, IL; 1:100). DAPI (Invitrogen Corporation, Carlsbad, CA) was used to detect nuclei.

### Image Analysis

For quantitative image analysis, serial coronal sections taken from 1.70 to 3.16 mm posterior to the bregma were examined for CD45^+ ^microglia and GFAP^+ ^astrocytes. Six animals from each group were evaluated. For each animal, antigens were detected in 5 parallel multi-brain sections having a defined distance of 160 μm and showing both the hippocampus and cortex. In each section, the hippocampus and the cortex were evaluated for the number of stained cells, and integral staining density (sum of all individual optical densities of each pixel in the area being measured). Images were acquired as digitized tiff files to retain maximal resolution using a Leica DMI 6000 B microscope microscope at 100 × final magnification with an attached digital camera system (Leica DFC 340 FX, Heerbrugg, Germany). The digital images were then routed into a Windows PC for quantitative analyses using Image-Pro Plus software, with the examiner blinded to sample identifiers. To maintain consistency across animals, a rectangular box (0.42 × 0.26 mm) was centered over the area of interest. Except for fewer sections used, all other quantifying procedures were the same as stereological quantification. The final results were shown as number of immunopositive cells or immunoreactivity per mm^2^.

### Western blot

At euthanasia, the cold PBS-perfused right hemispheres were further dissected into anterior cortex, posterior cortex, striatum, hippocampus, brainstem, and cerebellum. Protein extract preparation, total protein content determination, western blotting, and semi-quantitative analyses were performed as previously described [10,13]. Primary Abs used were: rabbit pAb to GRK5 (Santa Cruz Biotechnology, Santa Cruz, CA; 1:200) and mAb to β-actin (Sigma, St. Louis, MO; 1:1000).

### Statistics

Data were expressed as the mean ± S.E.M, and analyzed by ANOVA using Prism 4.3 (GraphPad Software, San Diego, CA). Post-hoc comparisons of means were made using Bonferroni's method with significance set at 0.05.

## Results

In order to determine whether GRK5 deficiency has an impact on brain inflammation in AD, we crossbred the TgAPPsw mice with the GRK5KO mice and generated the TgAPPsw mice deficient in GRK5. Only heterozygotes were used in this study, which included four genotype groups: WT control (APPsw^-/-^/GRK5^+/+^), GRK5KO heterozygote control (APPsw^-/-^/GRK5^+/-^), TgAPPsw (APPsw^+/-^/GRK5^+/+^) and the double (APPsw^+/-^/GRK5^+/-^) mice. As shown in Fig. 1, inactivation of one copy of the GRK5 gene resulted in at least 50% down-regulation of the GRK5 expression at the protein level in the heterozygote GRK5KO and the double mice.

**Figure 2:**
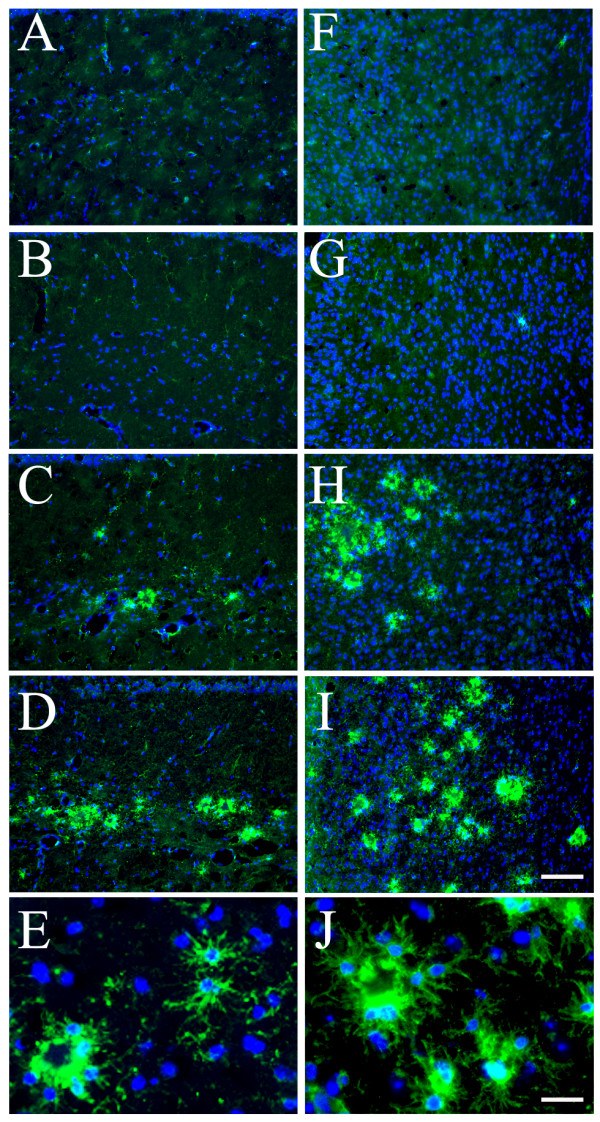
**Increased CD45^+ ^microglial cells in hippocampus and cortex of the TgAPPsw mice with reduced GRK5**. Representative IF results with anti-CD45 staining (green) in hippocampus **(A-D) **and cortex **(F-I) **of WT (**A **&**F**), heterozygote GRK5KO (**B **&**G**), TgAPPsw (**C **&**H**), and the double mice (**D **&**I**), respectively. Scale bar in panel **I **is for panels **A-D **and **F-I**: 100 μm. Panels **E **&**J**, examples of high magnification views of CD45^+^-microglial cells in hippocampus (**E**) and cortex (**J**) of the double mice that show details of activated microglial morphology. Scale bar in panel **J **is for panels **E **&**J**: 20 μm. Blue indicates reference DAPI staining of nuclei.

**Figure 3:**
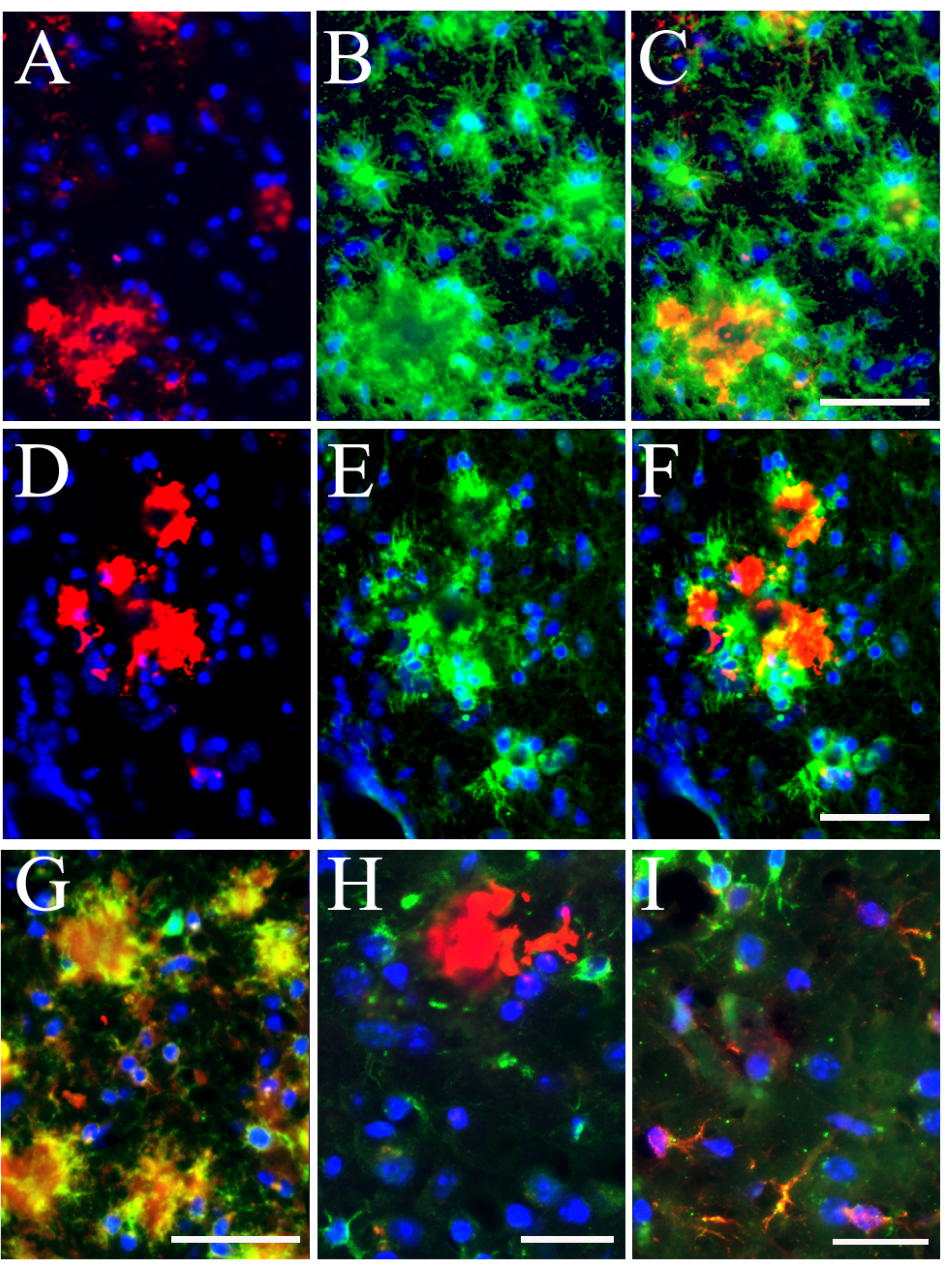
**Colocalization of activated microglial cells with Aβ plaques and IL-1β immunoreactivity in the TgAPPsw mice with reduced GRK5**. Panels **A-C**, IF staining of Aβ^+ ^plaques (**A**, red) and surrounding CD45^+ ^microglial cells (**B**, green), as well as their merged view (**C**) in the double mice. Scale bar: 50 μm for panels **A**-**C**. Panels **D-F**, IF staining of Aβ^+ ^plaques (**D**, red) and surrounding CD11b^+ ^microglial cells (**E**, green), as well as their merged view (**F**) in the double mice. Scale bar: 50 μm for panels **D**-**F**. Panel **G**, an example of merged view for CD45 (green) and CD11b/c (clone OX42, red) co-staining of the microglial cells. The image showed that, at least in this particular experimental paradigm, the CD45 antibody stained more specifically for the microglial cell profiles; while the OX42 antibody, in addition to its positive staining of the microglial cells, also non-specifically decorated the plaques. Scale bar: 45 μm. Panel **H**, an example of merged view for Aβ^+ ^plaques (red) and surrounding IL-1β immunoreactivity (green) in the double mice. Scale bar: 30 μm. Panel **I**, colocalization of CD45^+ ^microglial cells (green) with IL-1β immunoreactivity (red) in the double mice. Scale bar: 30 μm. Blue indicates reference DAPI staining of nuclei.

Evaluation of potential changes in brain inflammation was performed in 18-month old female mice, due to the known gender impact in these mice [18-20] [Li, 2008, BRB-submitted]. Immunofluorescent staining with antibodies against microglial and astrocyte markers was employed to reveal changes in microgliosis and astrogliosis. Our initial examination with antibody against CD45, a surface marker of microglia [15], revealed an increase of microgliosis in both hippocampal (Fig. 2A–D) and cortical (Fig. 2E–H) areas of the double mice as compared to the TgAPPsw mice. Subsequent characterization of the microgliosis showed that, consistent with previous findings [15], CD45^+ ^microglial cells were largely located surrounding or near Aβ deposits (Fig. 3A–C). Staining with a rat mAb to CD11b (Mac-1), another well-established microglial and macrophage marker [15,21], revealed similar changes of microglia in these animals (Fig. 3D–F). In addition, a mouse mAb OX-42 to CD11b/c also decorated the activated microglial cells in a way similar to anti-CD45 (Fig. 3G). However, at least in our experiments, OX-42 appeared to non-specifically stain the Aβ plaques, in addition to its positive staining for microglial cells. It has been previously established that activated microglial cells, at least at the early phase, produce IL-1β, an important proinflammatory cytokine that participates in a self-sustained vicious cycle to amplify the inflammatory neuronal damage [2]. Our examination revealed that, although less profound than CD45 immunoreactivity (IR), IL-1β-IR was indeed found near or surrounding the Aβ plaques (Fig. 3H) and was colocalized with CD45^+ ^microglial cells (Fig. 3I). Therefore, the microgliosis in the double mice displayed similar traits to that observed in other AD transgenic models, except for the exaggerated extent.

**Figure 4:**
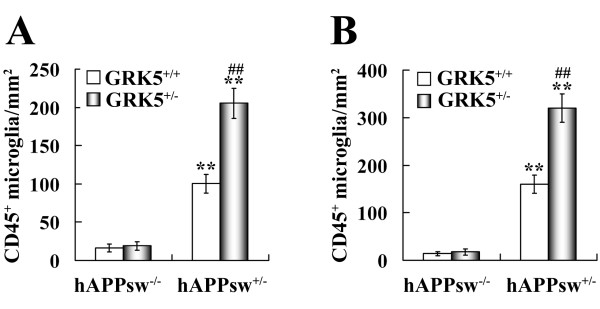
**Quantification of CD45^+ ^microglial cells in hippocampus and cortex of the TgAPPsw mice with reduced GRK5**. The numbers of CD45^+ ^microglial cells in hippocampus (**A**) and cortex (**B**) of WT, heterozygote GRK5KO, TgAPPsw, and the double mice were quantified as described in methods. Two way-ANOVAs revealed significant interactions between APPsw and GRK5 gene modifications for both hippocampus and cortex (P < 0.001 for both regions). The results for Bonferroni post-hoc comparisons of the means were shown as indicated. **P < 0.001 *vs *WT or heterozygote GRK5KO, ##P < 0.001 *vs *TgAPPsw mice.

To better understand the extent of the exaggerated microgliosis in these animals, we quantified CD45^+ ^microglial cells per mm^2 ^in hippocampus and cortex. Our quantitative image analysis (Fig. 4A &4B) showed that, as compared to the WT mice, both the TgAPPsw and the double mice displayed significantly more CD45^+ ^microglial cells in both of the examined brain regions (p < 0.001 for all); when compared to the TgAPPsw mice, the double mice had approximately double the number of CD45^+ ^microglial cells, in both hippocampus and cortex (P < 0.001). In addition, the heterozygote GRK5KO mice did not show any significant changes in the number of CD45^+ ^microglial cells, as compared to the WT, which is consistent with our previous report for the negative finding of brain inflammation in the GRK5KO mice [13]. When two-way ANOVA was used to analyze interactions between the two gene modifications (over-expression of human APPsw and inactivation of one copy of the murine GRK5 gene), a significance of p < 0.001 was revealed. Meanwhile, the changes caused by both gene modifications in the double mice were significantly higher than the sum of the changes caused by APPsw over-expression in TgAPPsw and by GRK5 gene inactivation in GRK5KO mice (p = 0.003 for hippocampus and p < 0.001 for cortex). These results indicate that the two gene modifications synergistically interact with each other, and both contribute the microgliosis observed in the double mice.

**Figure 5:**
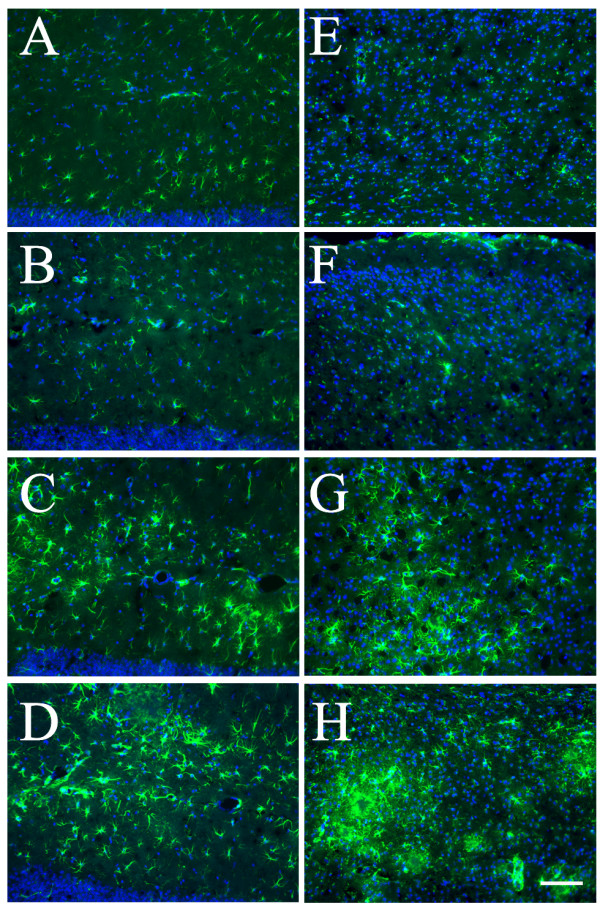
**Increased GFAP^+ ^astrocytes in hippocampus and cortex of the TgAPPsw mice with reduced GRK5**. Representative IF results with anti-GFAP staining (green) in hippocampus **(A-D) **and cortex **(E-H) **of WT (**A **&**E**), heterozygote GRK5KO (**B **&**F**), TgAPPsw (**C **&**G**), and the double mice (**D **&**H**), respectively. Blue indicates reference DAPI staining of nuclei. Scale bar: 100 μm.

**Figure 6:**
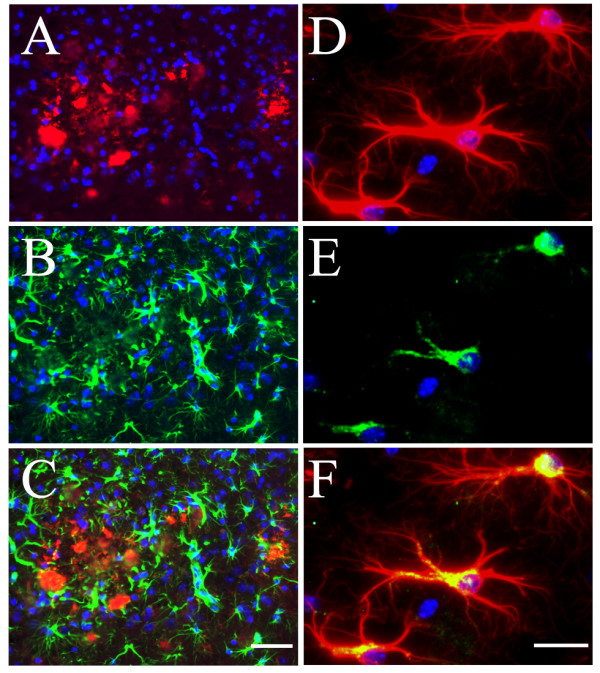
**Colocalization of reactive astrocytes with Aβ plaques and IL-6 immunoreactivity in the TgAPPsw mice with reduced GRK5**. Panels A-C, IF staining of Aβ^+ ^plaques (**A**, red) and nearby GFAP^+ ^reactive astrocytes (**B**, green), as well as their merged view (**C**) in the double mice. Scale bar: 50 μm for panels **A**-**C**. Panels **D-F**, IF staining of GFAP^+ ^astrocytes (**D**, red) and colocalized IL-6-IR (**E**, green), as well as their merged view (**F**) in the double mice. Scale bar: 20 μm for panels **D**-**F**. Blue indicates reference DAPI staining of nuclei.

**Figure 7:**
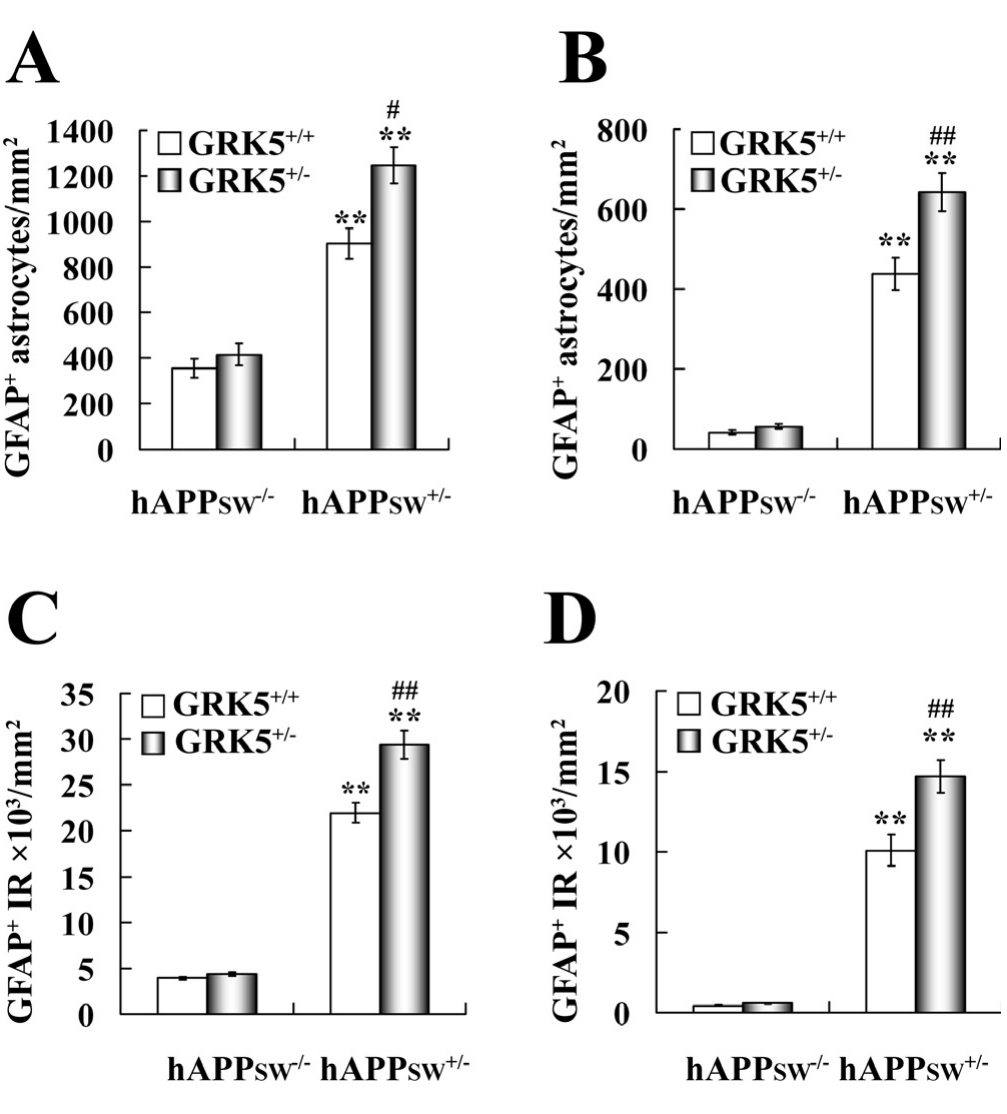
**Quantification of GFAP^+ ^astrocytes in hippocampus and cortex of the TgAPPsw mice with reduced GRK5**. Both the numbers of GFAP^+ ^astrocytes (**A **&**B**) and the intensity of GFAP-IR (**C **&**D**) in hippocampus (**A **&**C**) and cortex (**B **&**D**) of WT, heterozygote GRK5KO, TgAPPsw, and the double mice were quantified as described in methods. Two way-ANOVAs revealed significant interactions between APPsw and GRK5 gene modifications with the interaction factors of p < 0.05 for panel **A**, and p < 0.01 for panels **B**, **C**, and **D**. The results for Bonferroni post-hoc comparisons of the means were shown as indicated. **P < 0.001 *vs *WT or GRK5KO; #P < 0.01, ##P < 0.001 *vs *TgAPPsw mice.

In parallel to the microglial analysis, potential changes of astrogliosis in these animals were also assessed using IF staining with antibody to GFAP, an astrocyte-specific intermediate filament protein. We found more hypertrophic astrocytes brightly stained with anti-GFAP in both hippocampus and cortex from the double mice as compared to the TgAPPsw mice, and both the double mice and the TgAPPsw mice showed more hypertrophic astrocytes than the WT or heterozygous GRK5KO mice (Fig. 5A–H). Further characterization of the astrogliosis in the double mice revealed that although some of the hypertrophic astrocytes located near Aβ plaques (Fig. 6A–C), compared to the relation of microglia to Aβ plaques, hypertrophic astrocytes were more widespread in the entire brain region. In addition, at least some of the reactive astrocytes were IL-6 positive (Fig. 6D–F). Quantitative image analysis (Fig. 7A &7B) indicated that both the TgAPPsw and the double mice showed significantly more GFAP^+ ^astrocytes in the hippocampus and cortex (p < 0.001 for all) as compared to either the WT or the heterozygous GRK5KO mice; furthermore, the double mice displayed greater numbers of astrocytes than that in the TgAPPsw mice (p < 0.01 for hippocampus and p < 0.001 for cortex). Consistent with our previous report [13], the number of GFAP^+ ^astrocytes was not significantly different between the heterozygous GRK5KO and the WT mice. When two-way ANOVA was employed, significant interactions (p < 0.05 for hippocampus, and p < 0.01 for cortex) were revealed between the two gene modifications. The comparisons of effects of the double gene modifications with the sum of the effects of the each single gene modification alone revealed significant difference for the cortex (p = 0.02) but not for the hippocampus (p = 0.10).

It is known that both resting and reactive astrocytes express GFAP, but the latter usually expresses higher level of GFAP, and therefore stain brighter. Because of this, counting the number of GFAP^+ ^astrocytes may not accurately reflect the extent of the astrogliosis. Therefore, a quantitative image analysis of GFAP-IR intensity was also performed in parallel to the GFAP^+ ^cell counting. The results were similar to those from simple cell number counting, except that the significance was more striking (Fig. 7C &7D). The double gene modification effects were significantly higher than the sum of the each single gene alone for both hippocampus (p = 0.006) and cortex (p = 0.009). The interactions between the two gene modifications were also highly significant for both brain regions (p = 0.002 for hippocampus, and p = 0.005 for cortex). Therefore, these results suggest that Swedish mutated APP gene over-expression and GRK5 deficiency synergistically interact with each other to promote the astrogliosis observed in the double mice.

## Discussion

By analyzing the extent of gliosis in the brains of TgAPPsw mice with normal or reduced GRK5 expression, we demonstrated that the TgAPPsw mice with reduced GRK5 displayed significantly exaggerated microgliosis and astrogliosis. Consistent with our previous report [13], GRK5 deficiency alone does not lead to any significant inflammatory changes in either the heterozygote or homozygote GRK5KO mice. However, when an inflammation is present, as evoked by overexpression of the Swedish mutated APP gene, the GRK5 deficiency synergistically interacts with the active inflammatory processes and leads to amplified inflammatory responses. Such phenomena are consistent with the manner of GRK action, as GRKs are not thought to induce signals directly, but rather, they primarily modify the activity of activated GPCRs by uncoupling the receptor from G-protein, and initialing receptor internalization [11,12]. In the GRK5KO mice, the reason why we did not observe a significant inflammation is likely because there was no an active inflammatory signaling for the GRK5 to act on. In the double mice, however, when Swedish mutated APP was overexpressed, the accumulation of fibrillar Aβ initiated a complex of active inflammatory processes, which provided an opportunity to reveal impact of GRK5 deficiency. As demonstrated in this study, inactivation of one copy of the native GRK5 gene led to approximately doubled extent of microgliosis and significantly exaggerated astrogliosis. Therefore, these *in vivo *results strongly support the model that GRK5 deficiency indeed plays a significant role in amplifying the brain inflammatory responses in AD, as we previously speculated.

Since the primary role of GRKs is to desensitize GPCRs and dampen the activated GPCRs signaling, deficiency of GRK5 may lead to hyperactivity of those GPCRs that are specifically regulated by GRK5. As previously demonstrated, the fibrillar Aβ can initiate a complicated inflammatory process, which may include increased levels of proinflammatory cytokines, chemokines, and activated complement components [1-7]. It is known that receptors for all chemokines and the anaphylatoxin C3a and C5a are GPCRs [6,8,9]. In fact, even fibrillar Aβ itself has been shown to activate GPCRs, such as macrophage scavenger receptor, or formyl chemotactic receptor 2 (FPR2 or FPRL-1) [22-24]. Therefore, theoretically, all these GPCRs could be potential substrates for GRK5. On the other hand, however, the substrate spectrums for different GRK members may overlap, and the lost function due to deficiency of one GRK member may be largely compensated by other GRK members [11,12]. Only when the lost function cannot be fully compensated, a phenotype relevant to the specific GPCR signaling can then be revealed. For example, GRK2, GRK5, and GRK6 KO mice have been shown to display selectively impaired desensitization of adrenergic, muscarinic and dopaminergic receptors, respectively [17,25,26]. Unfortunately, to the best of our knowledge, information regarding the specific regulation of GRK5 on most, if not all, of the GPCRs involved in the fibrillar Aβ initiated inflammatory processes, particularly the *in vivo *data, is unavailable. Therefore, further investigations are warranted to understand the detailed molecular mechanisms by which the GRK5 deficiency exaggerates the brain inflammation in the TgAPPsw mice.

The main purpose of this study was to determine whether or not GRK5 deficiency *in vivo *contributes to exaggerate brain inflammation, and the results confirmed the positive role of GRK5 deficiency in the TgAPPsw mice. In the meantime, the fact that the double mice displayed significantly stronger inflammatory changes than those in the TgAPPsw mice makes the double mice a distinct model from the original TgAPPsw mice. It has been suggested that inflammatory responses in currently available APP transgenic mouse models, including the TgAPPsw mice, are not as strong as those in human AD patients [16]. Therefore, at least in resembling the strength of the inflammatory responses in AD, the TgAPPsw mice with reduced GRK5 may be a better model than the single TgAPPsw mice, and should serve as an additional AD animal model for relevant studies.

## Conclusion

This study has demonstrated that GRK5 deficiency *in vivo *significantly exaggerates the microgliosis and astrogliosis in the presence of an inflammatory initiator, the excess fibrillar Aβ and the subsequent active brain inflammatory reactions in the TgAPPsw mice. In the absence of an active inflammatory response, however, the effects of the GRK5 deficiency on inflammation *in vivo *remain silent. These findings, along with our previous findings for the role of GRK5 deficiency in promoting axonal defects and synaptic degeneration [13] [Li, 2008, BRB-submitted], strongly support that the hypothesis that GRK5 deficiency plays a significant role in AD pathogenesis. In addition, the TgAPPsw mouse combined with deficient GRK5 is a better model than the single TgAPPsw mouse, due to a stronger resemblance to the strength of the inflammatory responses in AD.

## Abbreviations

AD: Alzheimer's disease; Aβ: β-amyloid; GPCRs: G-protein coupled receptors; GRK5: GPCR kinase-5; GRK5KO: GRK5 knockout; βAPP: β-amyloid precursor protein; PCR: polymerase chain reaction; PBS: phosphate buffered saline; IF: Immunofluorescent; mAb: monoclonal antibody; pAb: polyclonal antibody; GFAP: glial fibrillary acidic protein; IL-1β: interleukin-1beta; IL-6: interleukin-6; IR, immunoreactivity.

## Competing interests

The authors declare that they have no competing interests.

## Authors' contributions

LL carried out the pathological studies, performed the statistical analysis, and participated in the maintenance of the transgenic mice and initial drafting of the manuscript, JL participated in the establishment and maintenance of the transgenic mouse colonies, WZS designed the study, supervised and coordinated the experimental conduction, and helped to draft the manuscript. All authors read and approved the final manuscript.
